# Two New *Ainsliaea* Species (Asteraceae: Pertyoideae) From Southeast China: Based on Morphological Characters and Phylogenetic Evidence

**DOI:** 10.1002/ece3.71088

**Published:** 2025-03-06

**Authors:** Cheng‐Yuan Zhou, Xiangxiu Su, Chunyi Wei, Liang Ma, Shi‐Pin Chen

**Affiliations:** ^1^ College of Forestry Fujian Agriculture and Forestry University Fuzhou China; ^2^ Key Laboratory of National Forestry and Grassland Administration for Orchid Conservation and Utilization at the College of Landscape Architecture Fujian Agriculture and Forestry University Fuzhou China; ^3^ Feng Yang Seedling Plantation Pingnan China; ^4^ Fujian Health College Fuzhou China

**Keywords:** Asteraceae, morphology, new species, phylogeny, plastid genome, taxonomy

## Abstract

Two new species of *Ainsliaea* (Asteraceae: Pertyoideae), *Ainsliaea erectifolia* X.X.Su and S.P.Chen and *Ainsliaea laxiflora* X.X.Su and S.P.Chen, both from Fujian, Southeast China, are described and illustrated here. Morphologically, *A. erectifolia* is similar to *A*. *simplicissima*, but can be distinguished by both surfaces of leaves being glabrous and the capitulum bearing 3 flowers. *A. laxiflora* is similar to *A. polystachya* , but differs by its rosette leaves aggregating near the middle part of the stem, the capitulum bearing 3 flowers, the terminal inflorescence rachilla shorter than lateral rachillae, anthers lower than corolla lobes, and style equal to or slightly higher than corolla lobes. Phylogenetic analyses indicated that the two new species were closely related to each other and belong to section *Aggregatae*. These two species have currently been assessed as Critically Endangered (CR) according to the IUCN guidelines. Furthermore, the complete plastid genomes of these two new species are reported. This study not only introduces two new *Ainsliaea* species but also provides their plastid genome data, offering valuable genetic resources for understanding the evolutionary history of the genus *Ainsliaea*.

## Graphical abstract

We have identified two new Ainsliaea species from China, Ainsliaea erectifolia X.X.Su & S.P.Chen and *Ainsliaea laxiflora* X.X.Su & S.P.Chen. Both the morphological observation and phylogenetic investigation support the status of Ainsliaea erectifolia and Ainsliaea laxiflora as new species. Besides, we reported four Ainsliaea plastomes from two new species.

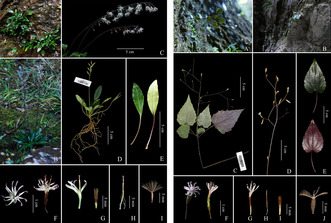

## 1 Introduction

*Ainsliaea* Candolle (1838: 13) is the largest genus of subfamily Pertyoideae in the family Asteraceae, comprising approximately 50 species (Zhang et al. 2019; Zhang et al. 2021). This genus is mainly distributed in Southeast Asia, with a few species extending to Malesia, the Philippines, and Indonesia (Freire 2007; Mitsui et al. 2008). China is the biodiversity center of this genus, comprising more than 40 species, with approximately 30 endemic species (Freire 2007; Zhang et al. 2019; Zhang et al. 2021). This genus is characterized by the unbranched stem, leaves rosulate or arranged at the middle of the stem, capitula in spikes, racemes, or panicles, florets few, corollas deeply 5‐lobed, and a plumose pappus (Zhang et al. 2024).

Morphological classifications of *Ainsliaea* mainly relied on habit types and leaf arrangements since its establishment (de Candolle 1838). Based on different leaf arrangements, Beauverd (1909) divided *Ainsliaea* into three sections (*A*. sect. *Scaposae* Beauverd, *A*. sect. *Aggregatae* Beauverd and *A*. sect. *Frondosae* Beauverd). However, the strong subjectivity of morphological classifications has led to many controversies on species definitions and infrageneric classifications within this genus (Beauverd 1909; Kitamura 1940; Tseng 1996; Freire 2007). Based on three molecular markers (ITS, ETS, and *ndhF*), Mitsui et al. (2008) showed significant discordances between morphology‐based classification and molecular phylogeny, and concluded that the sections defined by Beauverd (1909) were non‐monophyletic. Recently, Zhang et al. (2024) proposed the most comprehensive phylogenetic study of *Ainsliaea*, dividing the genus into two subgenera and four sections based on a combination of morphological and molecular evidence. This study significantly improves our understanding of *Ainsliaea* taxonomy and systematics.

During our botanical exploration to Yongtai County in eastern Fujian Province, China, in 2017 and 2020, two unknown *Ainsliaea* species were discovered, which differed from any known species. For further morphological observation, several individuals of the two species were introduced and cultivated at Fujian Agriculture and Forestry University (Fuzhou, Fujian). After a thorough literature review, morphological examination, and phylogenetic analyses, we found that these two *Ainsliaea* species do not match any previously known taxa. Here, we describe them as new species and use DNA sequences to confirm their phylogenetic positions, naming them *Ainsliaea erectifolia* X.X.Su & S.P.Chen and *Ainsliaea laxiflora* X.X.Su & S.P.Chen, respectively. Additionally, the complete plastid genomes of these two new species were first assembled, annotated, and employed to reconstruct phylogenetic relationships, providing a valuable genetic resource for further studies.

## 2 Materials and Methods

### 2.1 Morphological Description

Specimens were collected from Yongtai County, Fujian Province, China. Morphological character descriptions and measurements of each species were obtained during fieldwork. Voucher specimens were collected and deposited at the herbarium of the Forestry College of Fujian Agriculture and Forestry University (FJFC). The description of floral anatomy was studied under a Nikon SMZ18 stereomicroscope.

### 2.2 Taxon Sampling and Sequencing

Fresh leaves from two individuals (from different populations) of each new species were collected and dried in silica gel. Total DNA was extracted using the cetyltrimethylammonium bromide (CTAB) method (Allen et al. 2006). Total genomic DNA was sequenced on the BGI (the Beijing Genomics Institute, Shenzhen, China) sequencing platform DNBseq‐T7, generating 150‐bp paired‐end reads. More than 20 Gb of raw data were generated for each individual. Quality control for raw data was performed by FastQC v.3.0 (Andrews 2010) using default parameters.

### 2.3 Plastome Assembly and Annotation

The complete plastomes were de novo assembled using the GetOrganelle pipeline (Jin et al. 2020) with default parameters. The resulting filtered graphs were checked by Bandage (Wick et al. 2015). The Plastid Genome Annotator (PGA) software (Qu et al. 2019) was employed to perform the annotation of complete plastid genomes, and the published sequence of *Ainsliaea henryi* (PP175243) was used as a reference. The annotated plastomes were manually adjusted in Geneious R11.1.5 (Kearse et al. 2012). The annotation circle maps were drawn by OGDRAW (Greiner et al. 2019).

### 2.4 Phylogenetic Analyses

Following Zhang et al. (2024), three DNA sequences, including nuclear ribosomal DNA (ITS and ETS) and a plastid sequence (*ndhF*), were selected as phylogenetic markers in this study. The nuclear DNA sequences (ITS, and ETS) of each new species were assembled using the Hybpiper package (Johnson et al. 2016), with published sequences of *Ainsliaea simplicissima* (MN38442 and MN384416) as the reference sequences. The *ndhF* sequences were extracted from the annotated plastomes using Geneious R11.1.5.

To confirm the phylogenetic position of these two new species, a total of 41 *Ainsliaea* taxa and four *Pertya* taxa were chosen as the ingroup and outgroup, respectively, based on a previous study (Zhang et al. 2024). A total of 37 sequences of *ndhF*, 41 of ITS, and 41 of ETS were downloaded from GenBank (Table 1). A total of six plastome sequences were downloaded from GenBank to reconstruct the phylogenomic relationship (Table 2). The DNA sequences were aligned by MAFFT (Katoh et al. 2002) with auto parameters. The PhyloSuite (Zhang et al. 2020) script “Concatenate Sequence” was used to combine the DNA sequences into a concatenated matrix. The phylogenetic trees were performed by maximum likelihood (ML), maximum parsimony (MP), and Bayesian inference (BI). ML analyses were conducted with IQ‐TREE (Nguyen et al. 2014), and the best substitution model was automatically identified by ModelFinder using the Bayesian Information Criterion (BIC) (Kalyaanamoorthy et al. 2017). For the MP analysis, we performed a heuristic search with 1000 random taxon stepwise addition sequences and tree‐bisection‐reconnection (TBR) branch swapping (Swofford and Sullivan 2003). For the BI analysis, we used MrBayes v. 3.2.7a (Ronquist et al. 2012) under the GTR + I + Γ substitution model. The Markov chain Monte Carlo (MCMC) algorithm was run for 10,000,000 generations, with one tree sampled every 100 generations. The first 25% of trees were discarded as burn‐in to construct majority‐rule consensus trees and estimate posterior probabilities (PPs).

**TABLE 1:** Scientific names, voucher information, and GenBank accession numbers (ITS, ETS and *ndhF*) of the specimens used in this study. A dash (−) indicates missing data.

Scientific name	Voucher	ITS	ETS	*ndhF*
*Ainsliaea acerifolia* Sch. Bip.	A. Fujisaki 93–3353	AB288427	AB288469	AB288510
*A. acerifolia* var. *subapoda* Nakai	H. Setoguchi JP2448	AB288428	AB288470	AB288511
*A. apiculata* Sch.Bip	H. Setoguchi JP2438	AB288429	AB288471	AB288512
*A. apiculata* var. *acerifolia* Masam.	H. Setoguchi JP2434	AB288430	AB288478	AB288513
*A. aptera* DC.	H. Tabata 20,758	AB288431	AB288472	—
*A*. apteroides (C.C.Chang) Y.C.Tseng	H. Chuang 0731465	AB288432	AB288473	AB288514
*A. cordifolia* Franch. et Savat.	H. Setoguchi JP2446	AB288433	AB288474	AB288515
*A. dissecta* Franch. et Savat.	T. Kobayashi 32,365	AB288434	AB288475	AB288516
*A. elegans* var. *strigosa* Mattf.	H. Chuang 0731544	AB288435	AB288476	AB288517
*A. foliosa* Hand.‐Mazz.	H. Chuang 3589	AB288437	AB288479	—
*A. fragrans* Champ. ex Benth	Y. Mitsui C06801	AB288438	AB288480	AB288519
*A. fragrans* var. *integrifolia* (Maxim.) Kitam.	H. Setoguchi JP 2462	AB288439	AB288481	AB288520
*A. fulvipes* Jeffrey et w. w. Sm.	H. Chuang 1514	AB288440	AB288482	AB288521
*A. glabra* Hemsl.	B. L. Chen 0731626	AB288441	AB288483	AB288522
*A*. *gongshanensis* H.Chung	H. Chuang 0731637	AB288442	AB288484	AB288523
*A. gracilis* Franch.	Y. Mitsui C06850	AB288443	AB288485	AB288524
*A*. *grossedentata* Franch.	H. Chuang 16,883	AB288444	AB288486	AB288525
*A. henryi* Diels	Y. Fei 0731686	AB288445	AB288487	AB288526
*A*. *heterantha* Hand.‐Mazz.	S. Chen, H. Setoguchi 20,006	AB288446	AB288488	AB288527
*A. lancifolia* Franch.	W. H. Wang 8078	AB288447	AB288489	—
*A. latifolia* (D. Don) Sch. Bip.	H. Setoguchi C200605	AB288448	AB288490	AB288528
*A. linearis* Makino	H. Setoguchi JP2436	AB288436	AB288477	AB288518
*A*. *ljiagngensis* H. Chung	S. Chen, H. Setoguchi 20,005	AB288451	AB288494	AB288532
*A. macrocephala* (Mattf.) Y.C.Tseng	S. Chen, H. Setoguchi 2,004,241	AB288452	AB288493	AB288533
*A*. *macroclinidioides* Hayata	H. Setoguchi 04 T‐M2311	AB288454	AB288495	AB288534
*A*. *macroclinidioides* var. *okinawaensis* (Hayata) Kitam.	H. Setoguchi JP2605	AB288455	AB288496	AB288535
*A. nana* Y.C.Tseng	Y.H. Cheng in G.W. Hu HGW‐1492	ON229502	ON168941	ON191023
*A. oblonga* Koidz	H. Setoguchi JP2449	AB288457	AB288498	AB288537
*A*. *pertyoides* Franch.	H. Chuang 0731991	AB288458	AB288499	AB288538
*A. polystachya* X.X.Su & M.J.Zhang	X.X. Su SXX20201101‐1	MW829545	MW836815	MW836818
*A*. *simplicissima* M.J.Zhang & H.Q.Li	M.J. Zhang ZMJ20181116‐2	MN384420	MN384416	MN384418
*A. spicata* Vaniot	S. Chen, H. Setoguchi 2,004,092	AB288459	AB288500	AB288539
*A*. *sutchuenensis* Franch.	H. Chuang 0732127	AB288460	AB288501	AB288540
*A*. *trinervis* Y. C. Tseng	H. Chuang 0732148	AB288461	AB288502	AB288541
*A. uniflora* Sch.Bip.	H. Setoguchi JP2444	AB288468	AB288509	AB288547
*A. walkeri* Hook. f.	Z. Huang 604,507	AB288464	AB288503	—
*A. yunnanensis* Franch.	S. Chen, H. Setoguchi 2,004,137	AB288462	AB288504	AB288542
*A*. *erectifolia* X.X.Su & S.P.Chen_1	SXX0023_1	PQ651882	PQ659102	PQ653531
*A*. *erectifolia* X.X.Su & S.P.Chen_2	SXX0023_2	PQ651880	PQ659103	PQ653532
*A. laxiflora* X.X.Su & S.P.Chen_1	SXX0154_1	PQ651881	PQ659104	PQ653533
*A. laxiflora* X.X.Su & S.P.Chen_2	SXX0154_2	PQ651883	PQ659105	PQ653534
Outgroup				
*Pertya glabrescens* Sch. Bip.	T. Shimizu 11	AB288463	AB288505	AB288543
*P. rigidula* (Miq.) Makino	S. Yasuda 527	AB288465	AB288506	AB288544
*P. robusta* (Maxim.) Beauv.	S. Tsugaru, T. Takahashi 72,310	AB288466	AB288507	AB288545
*P. scandens* (Thunb. ex Murray) Sch. Bip.	H. Setoguchi JP2464	AB288467	AB288508	AB288546

**TABLE 2:** Scientific names, voucher information, and plastomes GenBank accession numbers of the specimens used in this study. A dash (−) indicates missing data.

Scientific name	Voucher	Accession number
*Ainsliaea gracilis* Franch.	DY159	OQ723680
*A. henryi* Diels	DY133	PP175243
*A. latifolia* (D. Don) Sch. Bip.	—	MW316662
*A*. *erectifolia* X.X.Su & S.P.Chen_1	SXX0023_1	PQ653531
*A*. *erectifolia* X.X.Su & S.P.Chen_2	SXX0023_2	PQ653532
*A. laxiflora* X.X.Su & S.P.Chen_1	SXX0154_1	PQ653533
*A. laxiflora* X.X.Su & S.P.Chen_2	SXX0154_2	PQ653534
Outgroup		
*Centaurea diffusa* Lam.	V236765	KJ690264
*Pertya multiflora* Cai F.Zhang & T.G.Gao	CHS2016003	MW148616
*P*. *phylicoides* Jeffrey	Z.X. Fu 4038	MN935435
*Saussurea polylepis* Nakai	SKK044232	MF695711

## 3 Results

### 3.1 Morphological Characters

Morphological observations indicate that *A*. *erectifolia* is similar to *A*. *simplicissima* M.J.Zhang & H.Q.Li (2019: 243), and *A. laxiflora* is close to *A. polystachya* X.X.Su & M.J.Zhang (2024: 277). However, *A*. *erectifolia* can be distinguished by leaves (both surfaces glabrous vs. both surfaces with very sparse villous pubescence), capitulum (bearing 3 flowers vs. bearing 1 flower) and flowering period (January to February vs. September to November) (Table 3). *A. laxiflora* can be differentiated by rosette leaves aggregated position (near the middle part of the stem vs. near the base or the lower part of the stem), capitulum (bearing 3 flowers vs. bearing 1 flower), terminal rachilla (shortest vs. longest), anthers (lower than corolla lobes vs. higher than corolla lobes), and style (equal or slightly higher than corolla lobes vs. greatly higher than corolla lobes) (Table 4).

**TABLE 3:** Morphological differences between *Ainsliaea erectifolia* and *A*. *simplicissima*.

Character	*A. erectifolia*	*A*. *simplicissima*
Leaf arrangement patterns	Rosette leaves arranged at base or at the lower part of the short stem	Rosette leaves arranged at base or at the lower part of the short stem
Leaf	Both surfaces glabrous	Both surfaces with very sparse villous pubescence
Capitula	Three flowers	One flower
Flowering period	January to February	September to November
Fruiting period	February to March	November to February

**TABLE 4:** Morphological differences between *Ainsliaea laxiflora* and *A. polystachya* .

Character	*A. laxiflora*	*A. polystachya*
Leaf arrangement patterns	Rosette leaves aggregated near the middle part of the stem	Rosette leaves aggregated near the base or the lower part of the stem
Capitula	Three flowers	One flower
Inflorescence shape	Terminal rachilla shorter than lateral rachillae	Terminal rachilla longer than lateral rachillae
Anther	4.0–5.0 mm long, lower than corolla lobes	4.0–5.0 mm long, higher than corolla lobes
Style	Extends out of anther tube, equal or slightly higher than corolla lobes, 11.0–12.0 mm long	Extends out of the anther tube, markedly higher than corolla lobes, ca. 11.0 mm long
Flowering period	June–October	October–November
Fruiting period	October–November	November–January

### 3.2 Characteristics of the Plastid Genome

**FIGURE 1:**
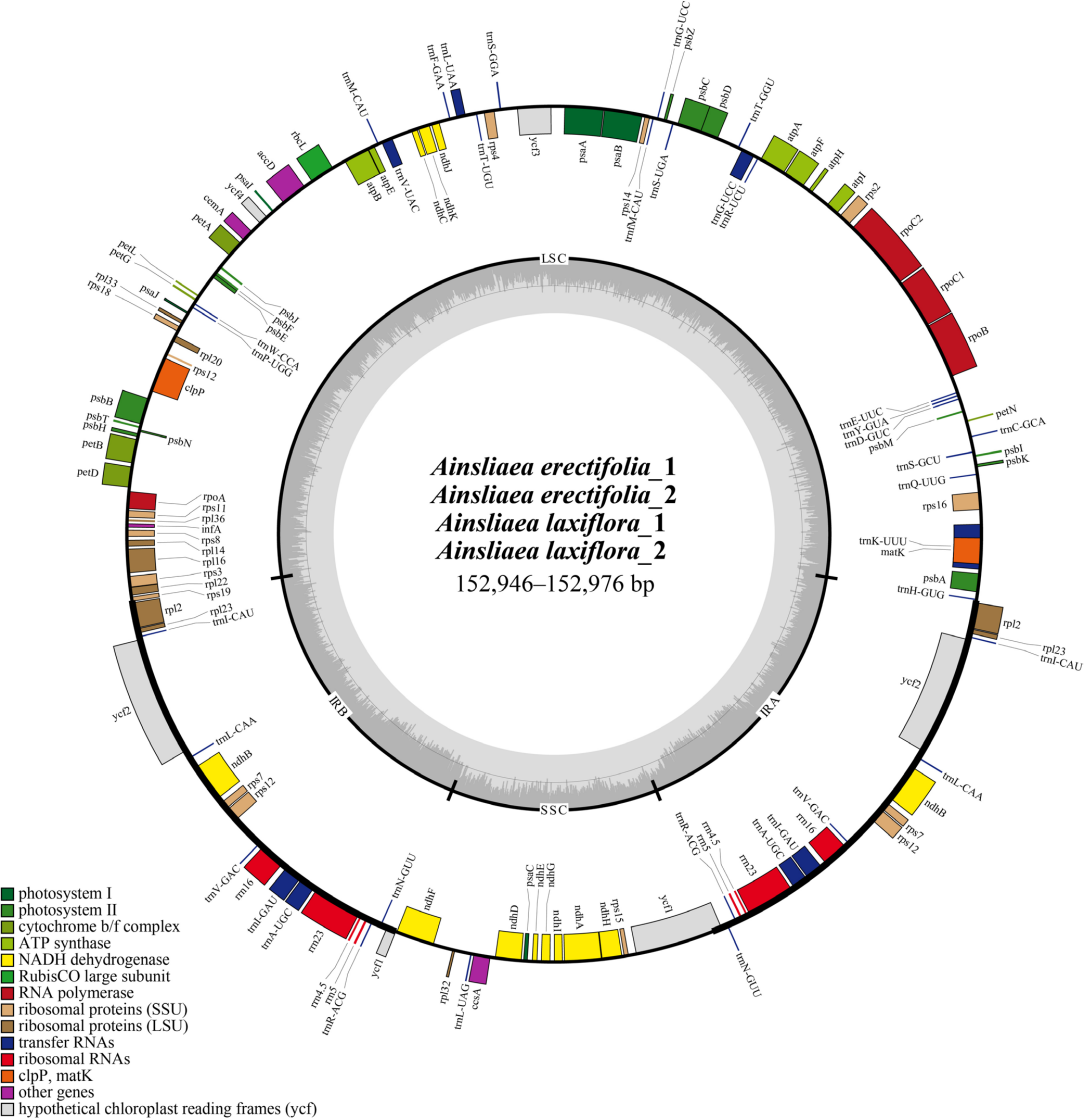
The plastome annotation map of *Ainsliaea erectifolia* and *Ainsliaea laxiflora*. The darker gray in the inner circle corresponds to GC content. The IRA and IRB (two inverted repeating regions); LSC (large single‐copy region); and SSC (Small single‐copy region) are indicated outside of GC content.

In this study, a total of four *Ainsliaea* plastomes were newly assembled and annotated. The plastomes of four individuals of two new *Ainsliaea* species displayed a typical quadripartite structure (Figure 1), consisting of a pair of IRs (25,198–25,200 bp), an LSC region (84,053–84,097 bp), and an SSC region (18,480–18,483 bp) (Table 5). Plastome sizes ranged from 152,946 to 152,976. The GC content of all these plastomes was 36.7%.

**TABLE 5:** Characteristics of the complete plastomes of *Ainsliaea erectifolia* and *A. laxiflora* .

Species name	Size (bp)	GC content (%)	LSC size in bp (%)	IR size in bp (%)	SSC size in bp (%)	Total number of gene	Protein‐coding gene	tRNA gene	rRNA gene	Number of pseudogenes
*A. erectifolia*_1	152,930	36.7	84,053 (54.96)	25,198 (16.48)	18,481 (12.08)	130	84	37	8	1
*A. erectifolia*_2	152,976	36.7	84,097 (54.97)	25,198 (16.47)	18,483 (12.08)	130	84	37	8	1
*A. laxiflora* _1	152,946	36.7	84,066 (54.96)	25,200 (16.48)	18,480 (12.08)	130	84	37	8	1
*A. laxiflora* _2	152,946	36.7	84,066 (54.96)	25,200 (16.48)	18,480 (12.08)	130	84	37	8	1

The plastomes of four newly sequenced *Ainsliaea* encoded 130 genes, including 84 protein‐coding genes, 37 transfer RNA (tRNA) genes, 8 ribosomal RNA (rRNA) genes, and one pseudogene (*ycf1* in IRB) (Table 5). Among these genes, 17 genes were replicated in the IR regions, comprising 6 protein‐coding genes (*rpl2*, *rpl23*, *rps7*, *rps12*, *ndhB* and *ycf2*), 4 rRNA genes (*rrn4*.5, *rrn5*, *rrn16*, and *rrn23*), and 7 tRNA genes (*trnA* ^*UGC*^, *trnI* ^*CAU*^, *trnI* ^*GAU*^, *trnL* ^*CAA*^, *trnN* ^*GUU*^, *trnR* ^*ACG*^, and *trnV* ^*GAC*^) (Figure 1).

### 3.3 Phylogenetic Relationships

The lengths of the aligned matrix of ITS, ETS and *ndhF* were 653 bp, 482 bp and 922 bp, respectively. The concatenated matrix of ITS, ETS and *ndhF* was 2057 bp, of which 405 (19.69%) were variable and 248 (12.06%) were parsimony informative. The best substitution model of the concatenated matrix was HKY + F + R2.

**FIGURE 2:**
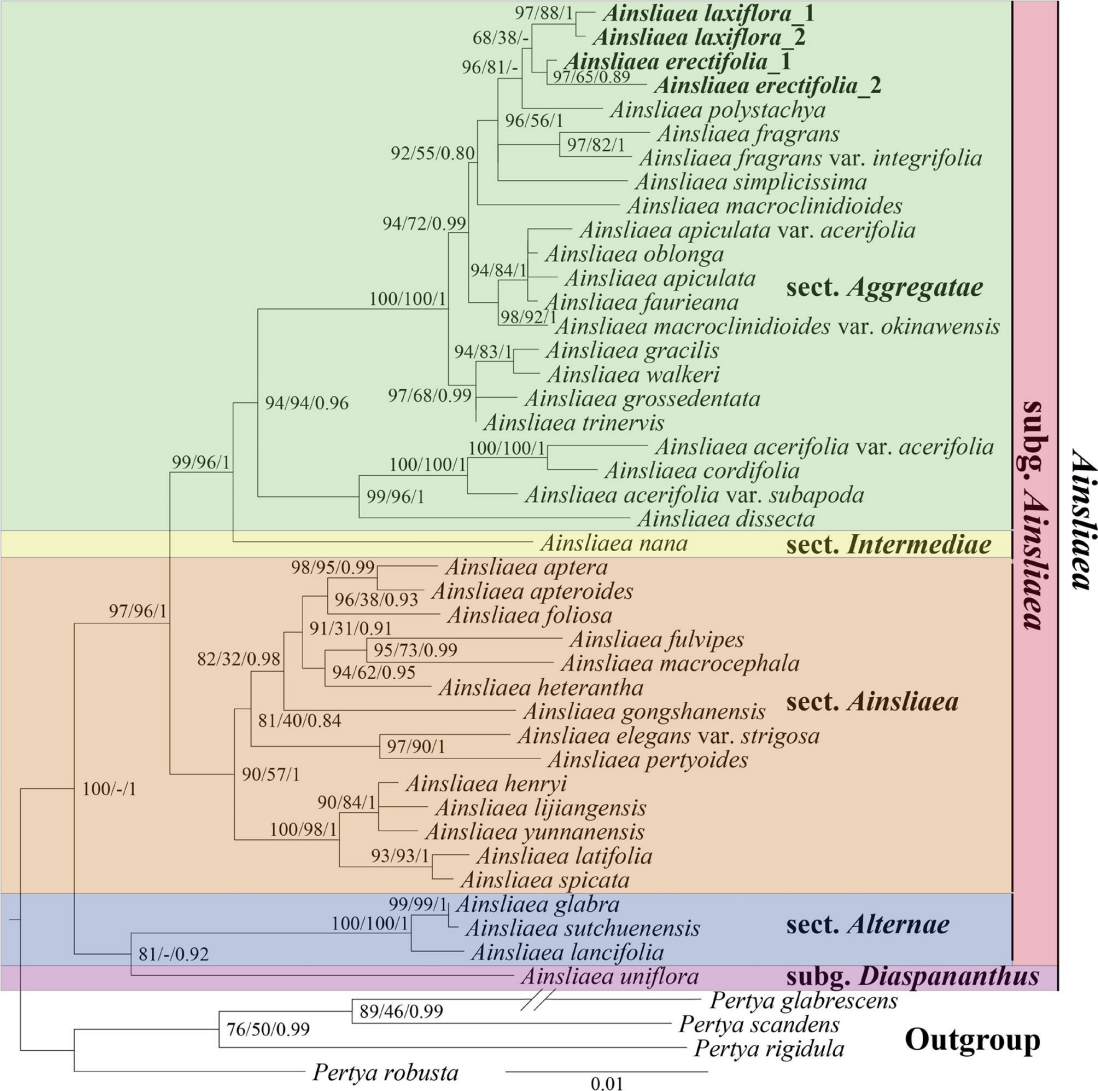
Phylogenetic tree obtained by ML analysis of the nuclear sequences (ITS and ETS) and *ndhF* combined matrix. The numbers near the nodes are bootstrap percentages and Bayesian posterior probabilities (BP_ML_, BP_MP_, PP). A dash (−) indicates that a node is inconsistent between the topology of the ML/MP trees and the Bayesian tree.

The present study reconstructed the phylogenetic relationships of the genus *Ainsliaea* using three methods (ML, MP and BI) based on three traditional molecular markers. A total of 41 *Ainsliaea* species were supported as a monophyletic, and the majority of the clades had moderate support (Figure 2). The intrageneric relationships of *Ainsliaea* showed that this genus could be divided into two clades with strong support. Both new species were supported as monophyletic taxa. In ML and MP analyses, the four individuals of two putative new species clustered into a clade, and both together were sisters to *A. polystachya* with strong support (BS_ML_ = 96, BS_MP_ = 81) (Figure 2). However, an incongruent phylogenetic relationship among these three species was detected in BI analysis. *A. polystachya* was embedded within the two putative new species with moderate support (PP = 0.54).

**FIGURE 3:**
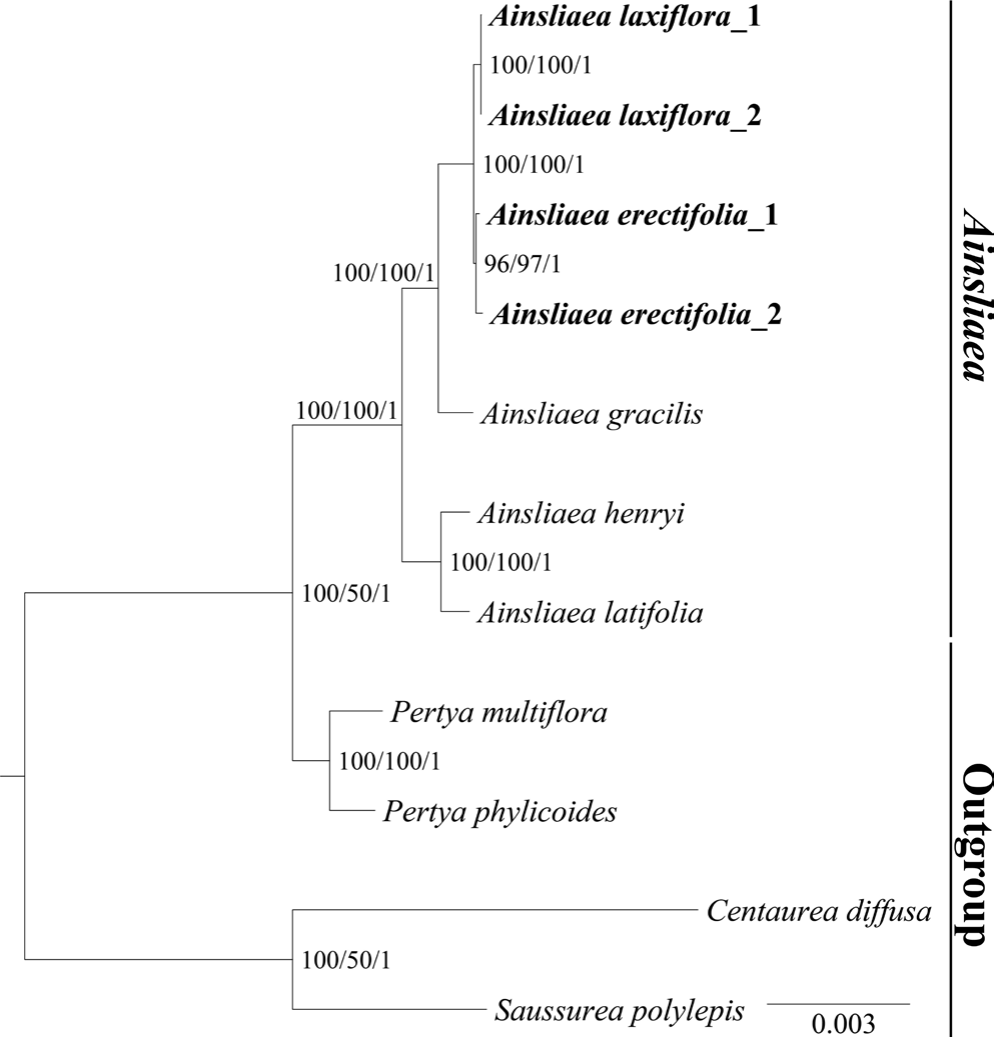
Phylogenetic tree obtained by ML analysis of the plastomes matrix. The numbers near the nodes are bootstrap percentages and Bayesian posterior probabilities (BP_ML_, BP_MP_, PP).

Additionally, based on complete plastome sequences, the phylogenetic analysis indicated that the *Ainsliaea* species formed a well‐supported monophyletic group (BS = 100, PP = 1.00), and the intrageneric relationships showed strong support (BS ≥ 96, PP = 1.00) (Figure 3). Two new *Ainsliaea* were sisters to each other, and both together are sisters to *A. gracilis* Franchet (1894: 297).

## 4 Discussion

The phylogenetic analyses presented here support *A. erectifolia* and *A. laxiflora* as monophyletic taxa, closely related to each other. These two new species were embedded within the section *Aggregatae*. According to the classification of Zhang et al. (2024), both *A. laxiflora* and *A*. *erectifolia* belong to sect. *Aggregatae* due to their leaves aggregated above the stem base, without scaly leaves on the lower stem. Thus, both morphological and molecular evidence support *A. laxiflora* and *A*. *erectifolia* as new species and belonging to section *Aggregatae*.

This study detected an incongruence between morphology and molecular analyses in *A. erectifolia* and *A. laxiflora* , which, despite their close phylogenetic relationship, exhibit significant morphological differences. This phenomenon has been reported in previous *Ainsliaea* phylogenetic studies (Zhang et al. 2024) and may be attributed to rapid radiation, reticulate evolution, and plastid capture (Drouin et al. 2008; Folk et al. 2016; Zhang et al. 2022). Additionally, apomixis is widely exhibited in the family Asteraceae, complicating the interpretation of phylogenetic trees (Noyes 2007). Further studies should utilize next‐generation sequencing (NGS) technology instead of traditional molecular markers to clarify the evolutionary history of *Ainsliaea*.

The genus *Ainsliaea* was presented as a taxonomically problematic group due to the complex habit type variations and extensive morphological diversity (Zhang et al. 2024). Previous phylogenetic studies revealed the monophyly of *Ainsliaea* and revised the classification according to phylogenetic results (Mitsui et al. 2008; Zhang et al. 2024). However, the interspecific relationships within *Ainsliaea* remain unclear due to conflicting topologies and moderate to weak support in numerous nodes (Zhang et al. 2024). Our present genus‐level phylogenetic results were consistent with previously published studies, supporting the monophyly status of *Ainsliaea*, with moderate support. The relatively weak phylogenetic resolution within this genus might be attributed to the lack of variable and informative sites.

One way to increase the resolution in phylogenetic trees is to employ molecular markers with more informative loci. Plastomes are suitable for reconstructing phylogenetic relationships in taxonomically problematic lineages due to their uniparental inheritance and abundance of informative loci (Sullivan et al. 2017; Li et al. 2024). However, to date, only three complete *Ainsliaea* plastomes ( *A. gracilis* , *A. henryi* Diels (1901: 628) and *A. latifolia* (D. Don) Sch. Bip. (1861: 190)) have been published in GenBank, extremely hindering our understanding of *Ainsliaea* phylogenetic relationships. Thus, in the present study, we report four *Ainsliaea* plastomes from two new species. These four plastomes had the typical quadripartite structure and showed similar plastome sizes and gene content to previously reported *Ainsliaea* plastomes (Chen et al. 2024), indicating that the *Ainsliaea* plastome may be highly conserved. Based on complete plastome sequences, the phylogenetic trees showed that the phylogenetic resolution within *Ainsliaea* has significantly improved with strong support (BS ≥ 96, PP = 1.00). This result indicates that plastome sequences are well‐suited for resolving the intrageneric relationships of *Ainsliaea* and provide valuable molecular resources for further phylogenetic studies.

## 5 Taxonomic Treatment

### 5.1 *Ainsliaea erectifolia* X.X.Su & S.P.Chen, *sp. nov.*

**FIGURE 4:**
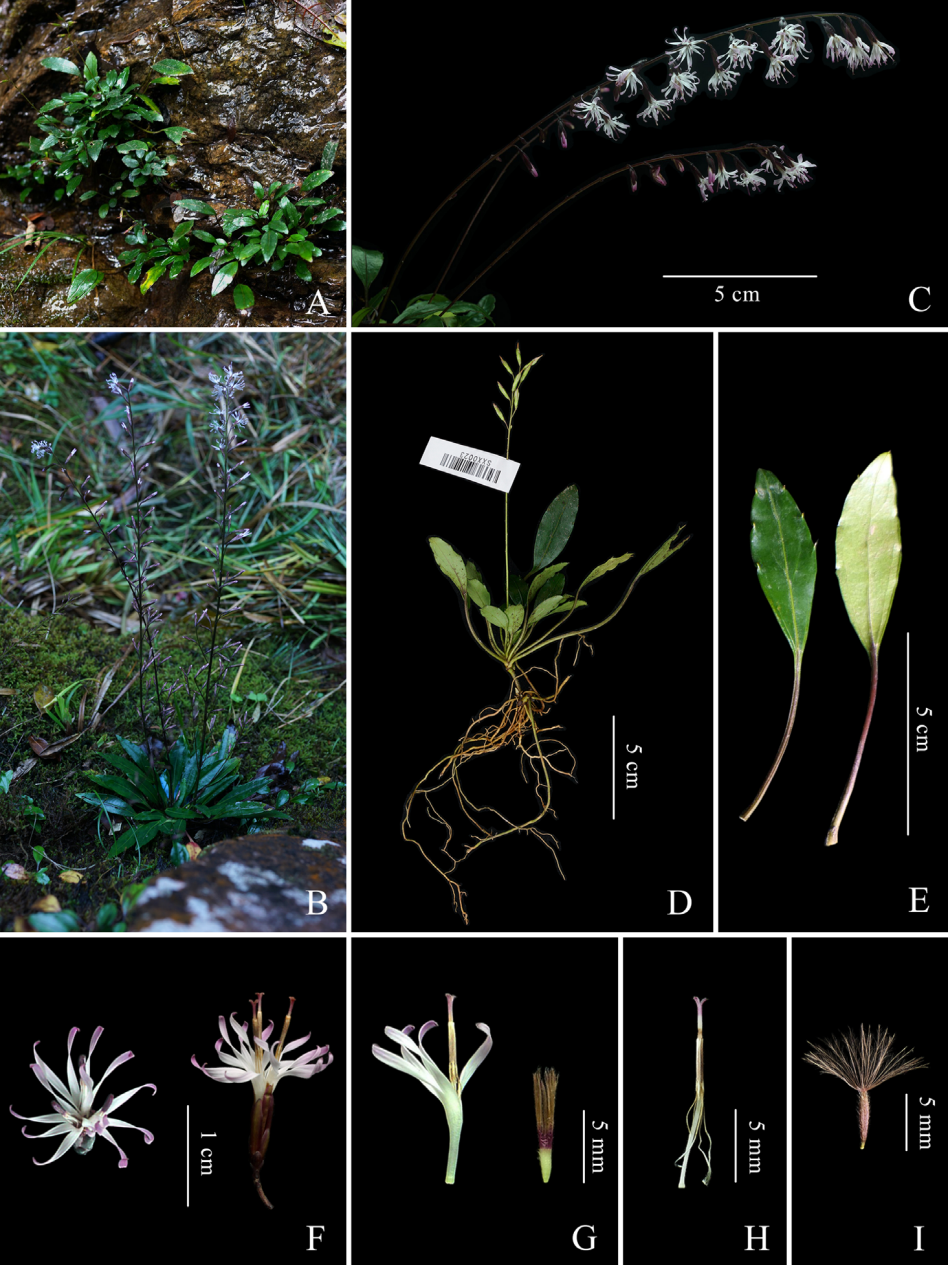
Habitat and morphology of *Ainsliaea erectifolia*. (A, B) Habitat. (C) Inflorescence. (D) Holotype. (E) Leaves (adaxial and abaxial surface). (F) Capitulum (front view and side view). (G) Flower. (H) Style and stigma with synantherous stamens. (I) Fruit.

**FIGURE 5:**
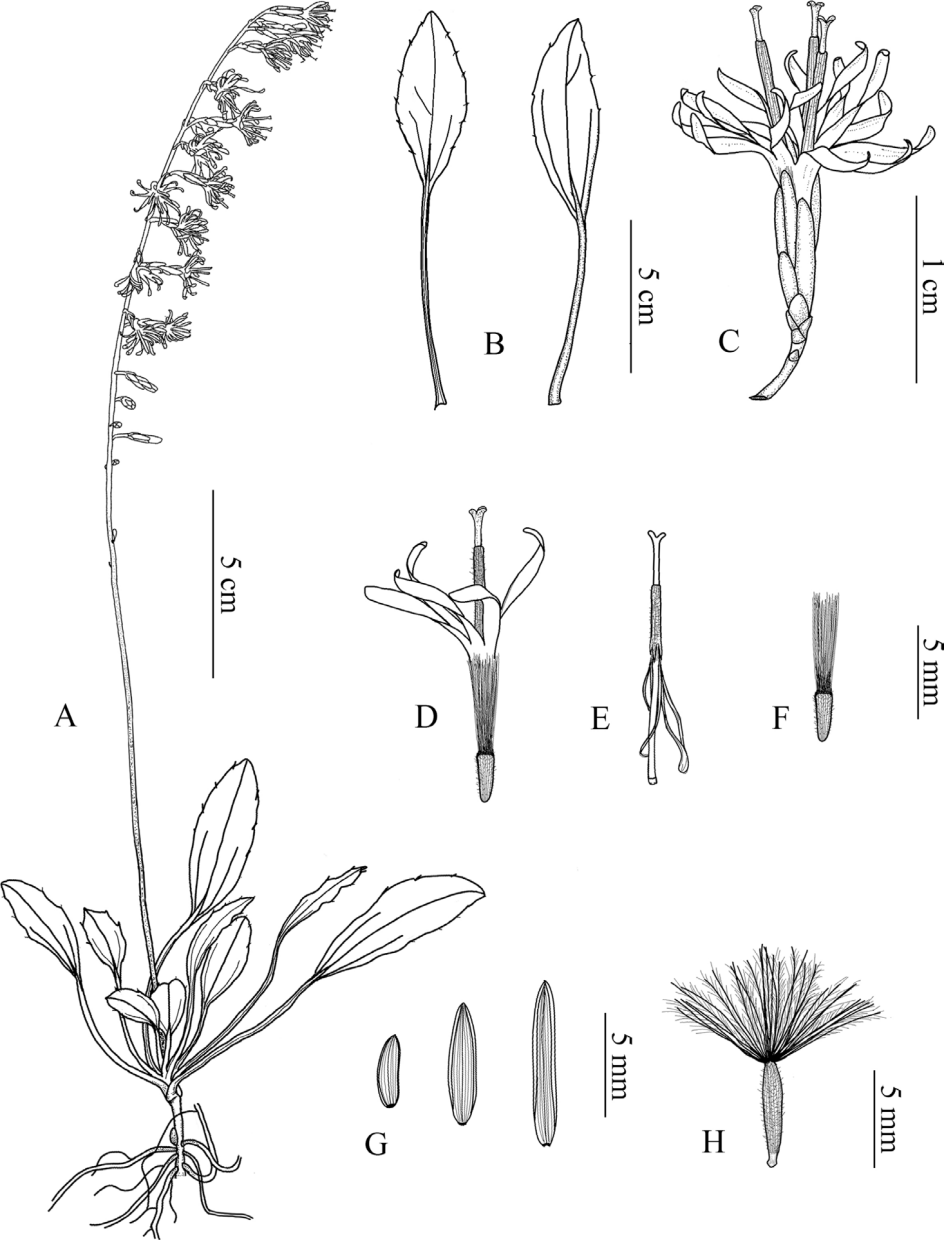
Line drawings of *Ainsliaea erectifolia*. (A) Plant. (B) Leaves (adaxial and abaxial surface). *C. capitulum* . (D) Flower. (E) Style and stigma with synantherous stamens. (F) Juvenile fruit. (G) Phyllary. (H) Fruit.

Type: CHINA. Fujian: Fuzhou, Yongtai, growing on wet slopes and rocks towards the valley, 25.73809° N, 118.85123° E, elev. ca. 200 m; 9 February 2022, voucher (SXX0261; SXXCS0261) (holotype, FJFC!; isotype, CSH!) (Figures 4 and 5).

### 5.2 Diagnosis

This species is morphologically similar to *Ainsliaea simplicissima* M. J. Zhang & H. Q. Li, from which it differs by the leaves both surfaces being glabrous and the capitulum bearing 3 flowers.

### 5.3 Description

Perennial herbs, 5.0–15.0 cm tall, with yellowish‐brown pubescence enveloping the new buds. Rhizome short, cylindrical, 1.0–3.0 mm in diameter, with 1–4 scale leaves. Roots numerous, fibrous. Stem erect, glabrous, 5.0–30.0 cm tall, 1.0–2.0 mm in diameter. Rosette leaves arranged at the base or at the lower part of the short stem, petiole equal to or longer than the blade; leaf blade papyraceous, elliptic or elliptic‐lanceolate, 2.0–6.0 cm long, 1.5–2.5 cm wide; apex acuminate, blade base attenuate and decurrent, margin with 2–5 obliquely upward‐slanting denticles each side from the middle to apex, adaxial surface dark green, abaxial surface pale green, both surfaces glabrous; basal veins 3, all veins slightly raised on both surfaces, midvein with sparse irregular lateral veins. Inflorescence arising from the center of the rosette leaves, erect, glabrous, 10.0–35.0 cm high, rachis glabrous. Capitula pedunculate, peduncle 1.0–4.0 mm, 3‐flowered, arranged in a unilateral raceme; involucre cylindrical, ca. 2 mm in diameter, 8.0–10.0 mm long; phyllaries 6–7‐seriate, glabrous, brown, outer phyllaries ovate, 1.0–2.0 mm, inner narrowly elliptic to lanceolate, ca. 6.0 mm. Florets bisexual; corollas white to lavender, tubular, ca. 10.0 mm, deeply 5‐lobed, lobes strip‐shaped, wavy, longer than corolla tube; anthers 5, extending out of the corolla tube, ca. 4.0 mm long, apical appendages truncate, base tails ca. 4.0 mm long; filament free, attached slightly below the apex of the corolla tube; style extends out of the anther tube, 11.0–13.0 mm long; stigmas 2, apices rounded or truncate; ovary long cylindrical, densely pubescent. Achenes terete, brown, densely hirsute, 4.0–5.0 mm long and ca. 1.0 mm in diameter; pappi free, dark or light yellow, plumose, ca. 8.0 mm long.

### 5.4 Phenology

Flowering period: January–February, Fruiting period: February–March.

### 5.5 Etymology

The species epithet refers to the first leaf of the plant being initially erect when it emerges.

### 5.6 Vernacular Name

Simplified Chinese: 竖叶兔耳风; Chinese pinyin: shù‐yè‐tù‐ěr‐fēng.

### 5.7 Distribution and Habitat

**FIGURE 6:**
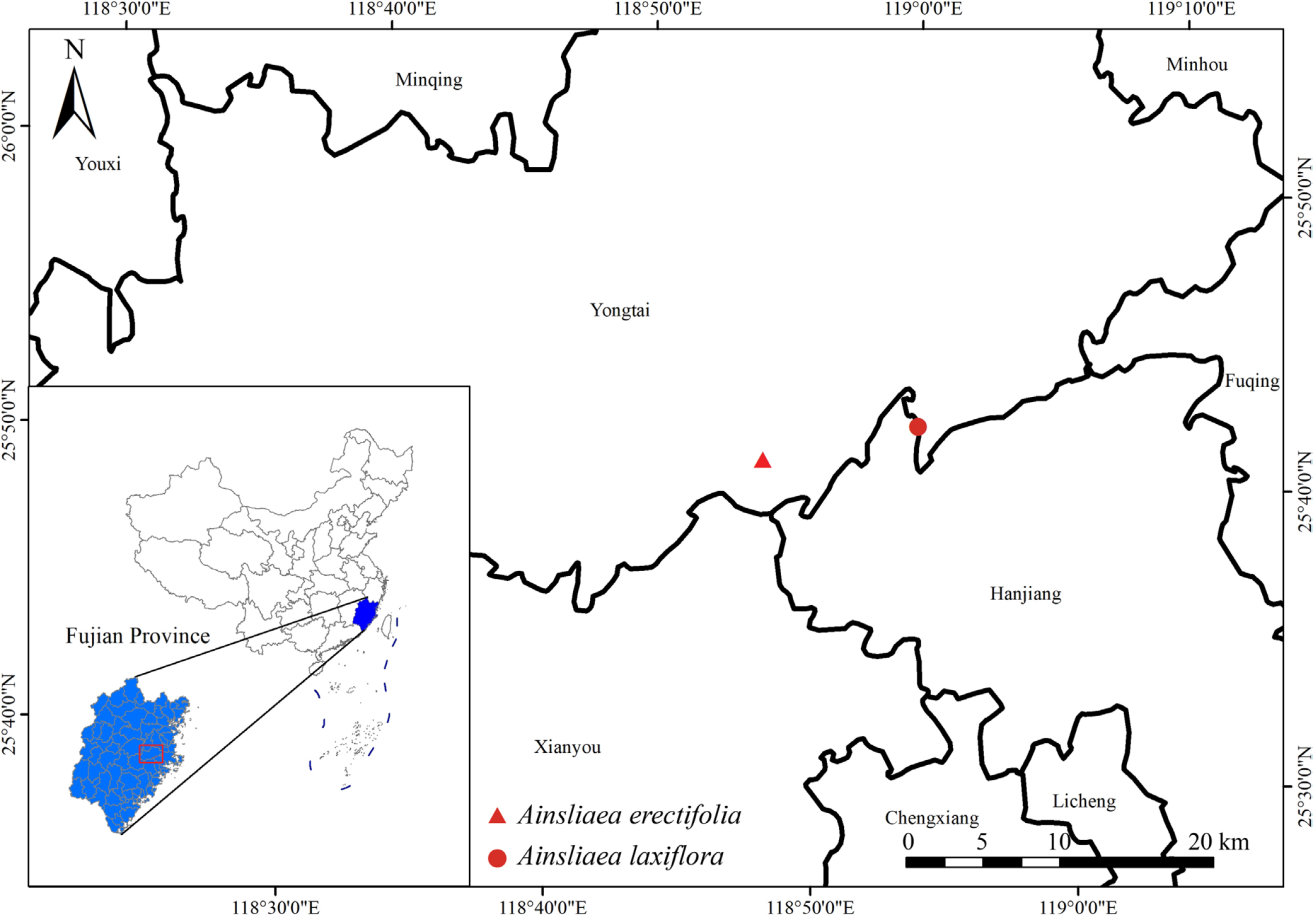
Distribution map of *Ainsliaea erectifolia* and *A. laxiflora* .

*Ainsliaea erectifolia* is so far only found within Yongtai, Fujian. This species grows on wet slopes and rocks towards the valley at elevations of 100–300 m (Figure 6). In this area, more than five populations have been found, each comprising approximately 10–20 individuals. Based on the current data, this species should be classified as Critically Endangered (CR) according to the IUCN guidelines (IUCN Standards and Petitions Committee 2022), due to its narrow distribution with only five populations with fewer than 100 plants.

### 5.8 *Ainsliaea laxiflora* X.X.Su & S.P.Chen, *sp. nov.*

**FIGURE 7:**
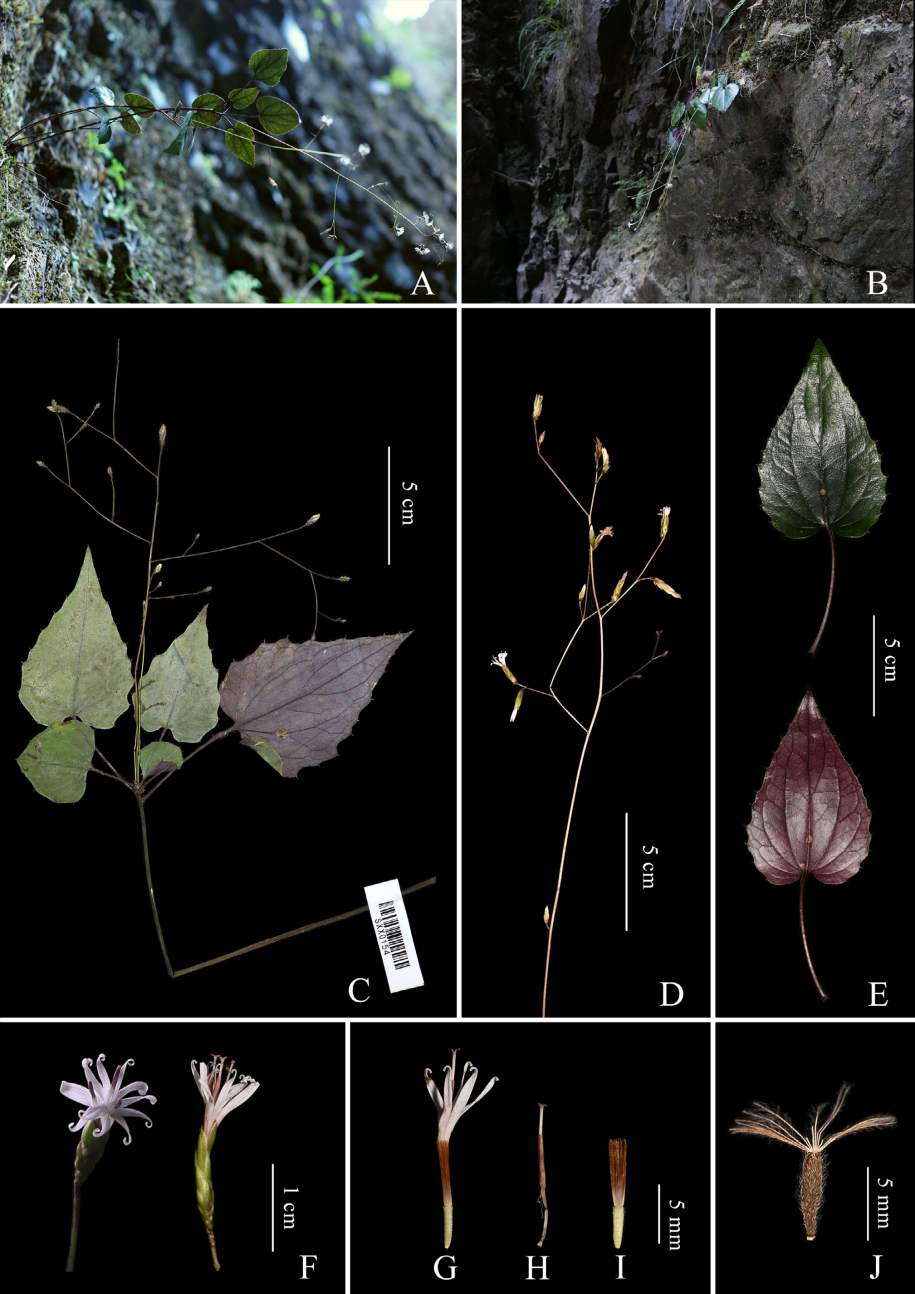
Habitat and morphology of *Ainsliaea laxiflora*. (A, B) Habitat. (C) Holotype. (D) Inflorescence. (E) Leaves (adaxial and abaxial surface). (F) Capitulum (front view and side view). (G) Flower. (H) Style and stigma with synantherous stamen. (I) Juvenile fruit. (J) Fruit.

**FIGURE 8:**
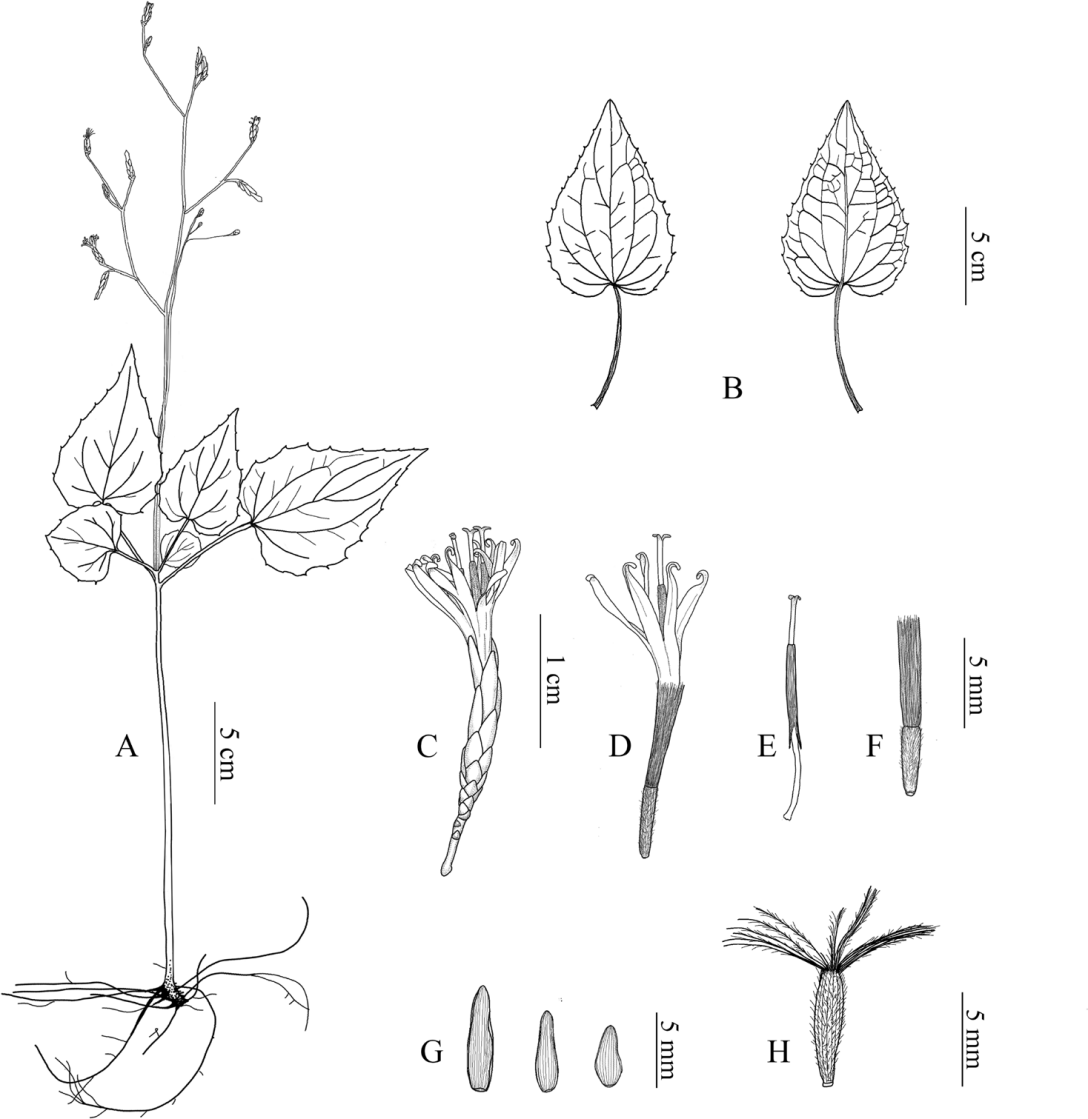
Line drawings of *Ainsliaea laxiflora*. (A) Plant. (B) Leaves (adaxial and abaxial surface). (C) *Capitulum* . (D) Flower. (E) Style and stigma with synantherous stamens. (F) Juvenile fruit. (G) Phyllary. (H) Fruit.

Type: CHINA. Fujian: Fuzhou, Yongtai, growing on wet slopes and rocks toward the valley, 25.74109° N, 118.95192° E, elev. ca. 500 m; 4 November 2017, voucher (SXX0311; SXXCS0311) (holotype, FJFC!; isotype, CSH!) (Figures 7 and 8).

### 5.9 Diagnosis

This species is similar to *Ainsliaea polystachya* X. X. Su & M. J. Zhang, but can be distinguished by its rosette leaves aggregated near the middle part of the stem, capitulum bearing three flowers, terminal inflorescence rachilla shorter than lateral rachillae, anthers lower than corolla lobes, and style equal to or slightly higher than corolla lobes.

### 5.10 Description

Perennial herbs, 15.0–35.0 cm tall. Roots numerous, slender, fibrous. Rhizome short, cylindrical, 3.0–5.0 mm in diameter, with 1–4 scale leaves. Rosette leaves aggregated near the middle part of the stem; petioles unequal, 2.0–6.0 cm, sparsely pubescent; leaf blade thickly chartaceous to thinly leathery, cordiform, ovate‐cordiform, 2.0–10.0 cm long, 2.0–8.0 cm wide; apex acuminate, base shallowly cordiform, margin with sparse acicular denticles, adaxial surface dark green, abaxial surface dark red to purple, sparsely pubescent on the veins and margin to glabrous; basal veins 3, lateral veins 2–3 pairs, all veins slightly raised on both surfaces. Inflorescence in open panicles, arising from the center of the leaves, 15.0–35.0 cm high, rachis glabrous, ebracteate. Capitula pedunculate, peduncle 1.0–3.0 cm, 3‐flowered, slim, terminal inflorescence rachilla shorter than lateral rachillae; involucre cylindric, ca. 2.5 mm in diameter, 6.0 mm long; phyllaries 6–8‐seriate, herbaceous, glabrous, green, with purple sides, outer phyllaries ovate, ca 2.0 mm, inner narrowly elliptic to lanceolate, ca. 5.0 mm. Florets bisexual; corolla white, tubular, ca. 8.0 mm long, deeply 5‐lobed, lobes wavy, slightly longer than the corolla tube; anthers 5, coalescent into a tube, 4.0–5.0 mm long, lower than the corolla lobes, apical appendages truncate, base tails ca. 4.0 mm long; filaments free, beard on the corolla throat, ca. 5.0 mm; style extends out of the anther tube, equal to or slightly higher than the corolla lobes, 11.0–12.0 mm long; stigmas 2, apices rounded or truncate; ovary long cylindrical, densely pubescent. Achenes subfusiform, brown, densely hirsute, 5.0–7.0 mm long and 1.0–2.0 mm in diameter; pappi free, dark yellow to brown, plumose, ca. 6.0 mm long.

### 5.11 Phenology

Flowering period: June–October, Fruiting period: October–November.

### 5.12 Etymology

The species epithet refers to its inflorescence, which is lax, with loose‐flowered.

### 5.13 Vernacular Name

Simplified Chinese: 疏花兔耳风; Chinese pinyin: shū‐huā‐tù‐ěr‐fēng.

### 5.14 Distribution and Habitat

During 7 years of fieldwork, we found that this species is, so far, only known from Yongtai, Fujian, where it grows on wet slopes and rocky areas near valleys at an elevation of approximately 500 m (Figure 7). Only two small populations have been found, with each consisting of fewer than 50 mature individuals. Its endangered status should be evaluated as CR (critically endangered), according to the IUCN (2022).

## Author Contributions

**Cheng‐Yuan Zhou:** data curation (lead), formal analysis (lead), investigation (supporting), methodology (lead), resources (supporting), software (lead), validation (lead), visualization (lead), writing – original draft (lead), writing – review and editing (lead). **Xiangxiu Su:** data curation (equal), investigation (lead), resources (lead). **Chunyi Wei:** data curation (equal), methodology (equal), software (supporting), visualization (supporting), writing – original draft (supporting), writing – review and editing (supporting). **Liang Ma:** data curation (equal), investigation (equal), resources (equal), supervision (supporting), visualization (equal). **Shi‐Pin Chen:** conceptualization (lead), funding acquisition (lead), project administration (lead), supervision (lead), writing – review and editing (equal).

## Conflicts of Interest

The authors declare no conflicts of interest.

## Data Availability

The DNA sequences generated in the present study have been deposited in the National Center for Biotechnology Information (NCBI) database. The accession numbers and the information on the voucher specimens are available in Table 1. The voucher specimens of the new species were housed in FJFC and CSH.

## References

[ece371088-bib-0001] Allen, G. C. , M. A. Flores‐Vergara , S. Krasynanski , S. Kumar , and W. F. Thompson . 2006. “A Modified Protocol for Rapid DNA Isolation From Plant Tissues Using Cetyltrimethylammonium Bromide.” Nature Protocols 1, no. 5: 2320–2325. 17406474 10.1038/nprot.2006.384

[ece371088-bib-0002] Andrews, S. 2010. FastQC: A Quality Control Tool for High Throughput Sequence Data. http://www.bioinformatics.babraham.ac.uk/projects/fastqc/.

[ece371088-bib-0003] Beauverd, G. 1909. “Contribution à l'étude des Composées Asiatiques.” Bulletin de la Société botanique de Genève 1: 364–388. https://www.biodiversitylibrary.org/page/5521049.

[ece371088-bib-0004] Chen, X. , Y. Feng , T. Qu , et al. 2024. “Complete Chloroplast Genomes of Two *Ainsliaea* Species and the Phylogenetic Analysis in the Tribe Pertyeae.” Frontiers in Genetics 15, no. 1: 1408114. 39109336 10.3389/fgene.2024.1408114 PMC11300268

[ece371088-bib-0005] de Candolle, A. P. 1838. Prodromus systematis naturalis regni vegetabilis. Vol. 7. sumptibus Sociorum Treuttel et Würtz. 10.5962/bhl.title.286.

[ece371088-bib-0006] Drouin, G. , H. Daoud , and J. Xia . 2008. “Relative Rates of Synonymous Substitutions in the Mitochondrial, Chloroplast and Nuclear Genomes of Seed Plants.” Molecular Phylogenetics and Evolution 49, no. 3: 827–831. 18838124 10.1016/j.ympev.2008.09.009

[ece371088-bib-0007] Folk, R. A. , J. R. Mandel , and J. V. Freudenstein . 2016. “Ancestral Gene Flow and Parallel Organellar Genome Capture Result in Extreme Phylogenomic Discord in a Lineage of Angiosperms.” Systematic Biology 66, no. 3: 320–337. 10.1093/sysbio/syw083 27637567

[ece371088-bib-0008] Freire, S. E. 2007. “Systematic Revision and Phylogeny of *Ainsliaea* DC. (Asteraceae, Mutisieae).” Annals of the Missouri Botanical Garden 94: 79–191.

[ece371088-bib-0009] Greiner, S. , P. Lehwark , and R. Bock . 2019. “OrganellarGenomeDRAW (OGDRAW) Version 1.3.1: Expanded Toolkit for the Graphical Visualization of Organellar Genomes.” Nucleic Acids Research 47, no. W1: W59–W64. 30949694 10.1093/nar/gkz238 PMC6602502

[ece371088-bib-0010] Jin, J. J. , W. B. Yu , J. B. Yang , et al. 2020. “GetOrganelle: A Fast and Versatile Toolkit for Accurate de Novo Assembly of Organelle Genomes.” Genome Biology 21, no. 1: 1–31. 10.1186/s13059-020-02154-5 PMC7488116 32912315

[ece371088-bib-0011] Johnson, M. G. , E. M. Gardner , Y. Liu , et al. 2016. “HybPiper: Extracting Coding Sequence and Introns for Phylogenetics From High‐Throughput Sequencing Reads Using Target Enrichment.” Applications in Plant Sciences 4, no. 7: 1–600016. 10.3732/apps.1600016 PMC4948903 27437175

[ece371088-bib-0012] Kalyaanamoorthy, S. , B. Q. Minh , T. K. F. Wong , A. von Haeseler , and L. S. Jermiin . 2017. “ModelFinder: Fast Model Selection for Accurate Phylogenetic Estimates.” Nature Methods 14: 587–589. 28481363 10.1038/nmeth.4285 PMC5453245

[ece371088-bib-0013] Katoh, K. , K. Misawa , K. Kuma , and T. Miyata . 2002. “MAFFT: A Novel Method for Rapid Multiple Sequence Alignment Based on Fast Fourier Transform.” Nucleic Acids Research 30: 3059–3066. 12136088 10.1093/nar/gkf436 PMC135756

[ece371088-bib-0014] Kearse, M. , R. Moir , A. Wilson , et al. 2012. “Geneious Basic: An Integrated and Extendable Desktop Software Platform for the Organization and Analysis of Sequence Data.” Bioinformatics 28: 1647–1649. 22543367 10.1093/bioinformatics/bts199 PMC3371832

[ece371088-bib-0015] Kitamura, S. 1940. “Compositae Japonicae, pars secunda.” Memoirs of the College of Science; Kyoto Imperial University. Series B. Biology 15, no. 3: 285–446.

[ece371088-bib-0016] Li, Y. , S. S. Zheng , T. R. Wang , et al. 2024. “New Insights on the Phylogeny, Evolutionary History, and Ecological Adaptation Mechanism in Cycle‐Cup Oaks Based on Chloroplast Genomes.” Ecology and Evolution 14, no. 9: e70318. 39290669 10.1002/ece3.70318 PMC11407850

[ece371088-bib-0017] Mitsui, Y. , S. T. Chen , Z. K. Zhou , C. I. Peng , Y. F. Deng , and H. Setoguchi . 2008. “Phylogeny and Biogeography of the Genus *Ainsliaea* (Asteraceae) in the Sino‐Japanese Region Based on Nuclear rDNA and Plastid DNA Sequence Data.” Annals of Botany 101: 111–124. 17981878 10.1093/aob/mcm267 PMC2701833

[ece371088-bib-0018] Nguyen, L. T. , H. A. Schmidt , A. von Haeseler , and B. Q. Minh . 2014. “IQ‐TREE: A Fast and Effective Stochastic Algorithm for Estimating Maximum‐Likelihood Phylogenies.” Molecular Biology and Evolution 32: 268–274. 25371430 10.1093/molbev/msu300 PMC4271533

[ece371088-bib-0019] Noyes, R. D. 2007. “Apomixis in the Asteraceae: Diamonds in the Rough.” Functional Plant Science and Biotechnology 1, no. 2: 207–222.

[ece371088-bib-0020] Qu, X. J. , M. J. Moore , D. Z. Li , and T. S. Yi . 2019. “PGA: A Software Package for Rapid, Accurate, and Flexible Batch Annotation of Plastomes.” Plant Methods 15: 50. 31139240 10.1186/s13007-019-0435-7 PMC6528300

[ece371088-bib-0021] Ronquist, F. , M. Teslenko , P. V. D. Mark , et al. 2012. “MrBayes 3.2, Efficient Bayesian Phylogenetic Inference and Model Choice Across a Large Model Space.” Systematic Biology 61, no. 3: 539–542. 22357727 10.1093/sysbio/sys029 PMC3329765

[ece371088-bib-0022] Sullivan, A. R. , B. Schiffthaler , S. L. Thompson , N. R. Street , and X.‐R. Wang . 2017. “Interspecific Plastome Recombination Reflects Ancient Reticulate Evolution in *Picea* (Pinaceae).” Molecular Biology and Evolution 34: 1689–1701. 28383641 10.1093/molbev/msx111 PMC5455968

[ece371088-bib-0023] Swofford, D. L. , and J. Sullivan . 2003. “Phylogeny Inference Based on Parsimony and Other Methods Using PAUP*.” In The Phylogenetic Handbook: A Practical Approach to Phylogenetic Analysis and Hypothesis Testing, edited by P. Lemley , M. Salemi , and A.‐M. Vandamme , 60–206. Cambridge University Press.

[ece371088-bib-0024] Tseng, Y. C. 1996. “Compositae (9): Mutisieae. 1103.” In Flora Reipublicae Popularis Sinicae, edited by Y. C. Tseng , vol. 79. Science Press.

[ece371088-bib-0025] Wick, R. R. , M. B. Schultz , J. Zobel , and K. E. Holt . 2015. “Bandage: Interactive Visualization of de Novo Genome Assemblies.” Bioinformatics 31, no. 20: 3350–3352. 26099265 10.1093/bioinformatics/btv383 PMC4595904

[ece371088-bib-0026] Zhang, C. F. , J. Tian , Y. H. Cheng , et al. 2024. “An Updated Phylogeny of *Ainsliaea* (Asteraceae: Pertyoideae) and Its Implications for Classification and Habit Evolution.” Taxon 73, no. 4: 1030–1052.

[ece371088-bib-0027] Zhang, D. , F. Gao , I. Jakovlić , et al. 2020. “PhyloSuite: An Integrated and Scalable Desktop Platform for Streamlined Molecular Sequence Data Management and Evolutionary Phylogenetics Studies.” Molecular Ecology Resources 20: 348–355. 31599058 10.1111/1755-0998.13096

[ece371088-bib-0028] Zhang, M. J. , X. X. Su , C. An , H. Q. Li , and Z. Zhang . 2021. “ *Ainsliaea Polystachya* (Asteraceae), a New Species From Fujian, China Based on Morphological and Molecular Evidence.” Phytotaxa 497, no. 3: 227–284.

[ece371088-bib-0029] Zhang, M. J. , W. J. Yu , and H. Q. Li . 2019. “ *Ainsliaea Simplicissima* (Asteraceae), a New Species From Southeast China and Its Phylogenetic Position.” Phytotaxa 424, no. 4: 243–252.

[ece371088-bib-0030] Zhang, Q. , L. Zhao , R. A. Folk , et al. 2022. “Phylotranscriptomics of Theaceae: Generic‐Level Relationships, Reticulation and Whole‐Genome Duplication.” Annals of Botany 129, no. 4: 457–471. 35037017 10.1093/aob/mcac007 PMC8944729

